# Bacterial Swarm-Mediated Phage Transportation Disrupts a Biofilm Inherently Protected from Phage Penetration

**DOI:** 10.1128/spectrum.00937-23

**Published:** 2023-06-26

**Authors:** Nichith K. Ratheesh, Amanda M. Zdimal, Cole A. Calderon, Abhishek Shrivastava

**Affiliations:** a Biodesign Center for Fundamental and Applied Microbiomics, School of Life Sciences, Center for Biological Physics, Arizona State University, Tempe, Arizona, USA; The Ohio State University Division of Biosciences

**Keywords:** biofilm, active transport, swarming, bacterial motility, collective motion, phage, oral microbiome, type 9 secretion system, gliding motility

## Abstract

Physical forces that arise due to bacterial motility and growth play a significant role in shaping the biogeography of the human oral microbiota. Bacteria of the genus *Capnocytophaga* are abundant in the human oral microbiota and yet very little is known about their physiology. The human oral isolate Capnocytophaga gingivalis exhibits robust gilding motility that is driven by the rotary type 9 secretion system (T9SS), and cells of *C. gingivalis* transport nonmotile oral microbes as cargo. Phages, i.e., viruses that infect bacteria, are found in abundance within the microbiota. By tracking fluorescently labeled lambda phages that do not infect *C. gingivalis*, we report active phage transportation by *C. gingivalis* swarms. Lambda phage-carrying *C. gingivalis* swarms were propagated near an Escherichia coli colony. The rate of disruption of the E. coli colony increased 10 times compared with a control where phages simply diffused to the E. coli colony. This finding suggests a mechanism where fluid flows produced by motile bacteria increase the rate of transport of phages to their host bacterium. Additionally, *C. gingivalis* swarms formed tunnel-like structures within a curli fiber-containing E. coli biofilm that increased the efficiency of phage penetration. Our data suggest that invasion by a *C. gingivalis* swarm changes the spatial structure of the prey biofilm and further increases the penetration of phages.

**IMPORTANCE** Dysbiosis of the human oral microbiota is associated with several diseases, but the factors that shape the biogeography of the oral microbiota are mostly opaque. Biofilms that form in the human supragingival and subgingival regions have a diverse microbial community where some microbes form well-defined polymicrobial structures. *C. gingivalis*, a bacterium abundant in human gingival regions, has robust gliding motility that is powered by the type 9 secretion system. We demonstrate that swarms of *C. gingivalis* can transport phages through a complex biofilm which increases the death rate of the prey biofilm. These findings suggest that *C. gingivalis* could be used as a vehicle for the transportation of antimicrobials and that active phage transportation could shape the spatial structure of a microbial community.

## INTRODUCTION

Phages are abundant in any microbial community, and typically, it is thought that they find their host through encounters whose probability depends on the rates of diffusion. Phages are promising alternatives to antibiotic treatment during chronic antimicrobial resistant (AMR) infections (1). However, phage therapy has several limitations that need to be resolved before it becomes a widely applied medical strategy. One limitation is that phages can get trapped in the matrix of infectious biofilms, which diminishes their frequency of encountering a bacterial cell (2, 3). Another limitation is that the larger size of phages than that of antibiotic molecules results in a slower diffusion rate. According to the Stokes-Einstein equation (4), the translation diffusion coefficient (*D*) of a sphere of radius *r* in a medium of viscosity η is *K_B_T*/6πη*r* where *K_B_* is the Boltzmann’s constant and T is the absolute temperature. *D* is proportional to the speed of diffusion, and the inverse relationship between *D* and *r* implies that small molecules diffuse faster. One of the largest known antibiotics, vancomycin (~1-nm radius), has 66 C atoms (5). The carbon chain lengths of commonly used medicinal antibiotics are about a quarter to half of vancomycin. For example, amoxicillin and cefalexin have 16 C atoms, ciprofloxacin and methicillin have 17 C atoms, and azithromycin has 38 C atoms (6–9). The capsid of the lambda phage used in our study has a radius of about 30 nm (10). This suggests that lambda phage will diffuse 30 times slower than vancomycin and 100 times slower than cefalexin. Additionally, it has been experimentally demonstrated that phage T4 and the filamentous phage fNEL diffuse slower than ciprofloxacin, penicillin, and tetracycline (11). To establish phage therapy as an efficient alternative to antibiotic treatment, one needs to also understand the forces that drive phage diffusion within a polymicrobial community.

Fluid flows are common within a microbial community, and they can provide a directionality to diffusion. One way to generate a directional fluid flow within a microbial community is through bacterial motility. Electron microscopy images have shown that phages bind to bacterial flagella (12), and an analysis of bacterial colonies suggests hitchhiking of phages (12, 13). While it appears that phages might be able to utilize the active motility of bacteria, live microscopy images of phages hitchhiking on bacteria are not available. Can one directly visualize phage transportation? Also, are motile but nonflagellated bacteria able to transport phages? Could such transportation shape a microbial community? We sought to answer these questions with the help of Capnocytophaga gingivalis, which is found in abundance in the human oral microbiota (14).

*C. gingivalis* exhibits gliding motility and transports nonmotile bacterial species of the human oral microbiota (15). Bacterial gliding is an active process, and individual rod-shaped gliding bacteria move in a screw-like fashion (16). Bacteria of the phylum *Bacteroidetes* are among the abundant members of the healthy human microbiome, and motile members of the phylum *Bacteroidetes* perform gliding motility with the help of the type 9 secretion system (T9SS) which is a rotary motor that couples with a cell-surface conveyor belt. SprB is loaded on the conveyor belt, and its interaction with an external substratum results in gliding motility (17, 18). On an agar surface, T9SS-driven cells of *C. gingivalis* and other gliding *Bacteroidetes* swarm in a vortex-like fashion (15, 19). Bacterial swarms are known to produce a wetting agent called the swarm fluid (20). The swarm fluid of *C. gingivalis* moves along the direction of the swarm (15). Here, we report that lambda phages are able to surf along the swarm fluid of a nonhost *C. gingivalis* swarm. This movement increases their diffusion rates by an order of magnitude and allows better penetration of a nonmotile Escherichia coli (host) colony. Moreover, a *C. gingivalis* swarm drills tunnels through a curli fiber-containing biofilm of E. coli. Due to a mesh of curli fiber, the E. coli biofilm is resistant to phage penetration. A combination of tunnel drilling and advective diffusion of phages renders a previously “phage-resistant” biofilm susceptible to phage infection.

## RESULTS

**FIG 1:**
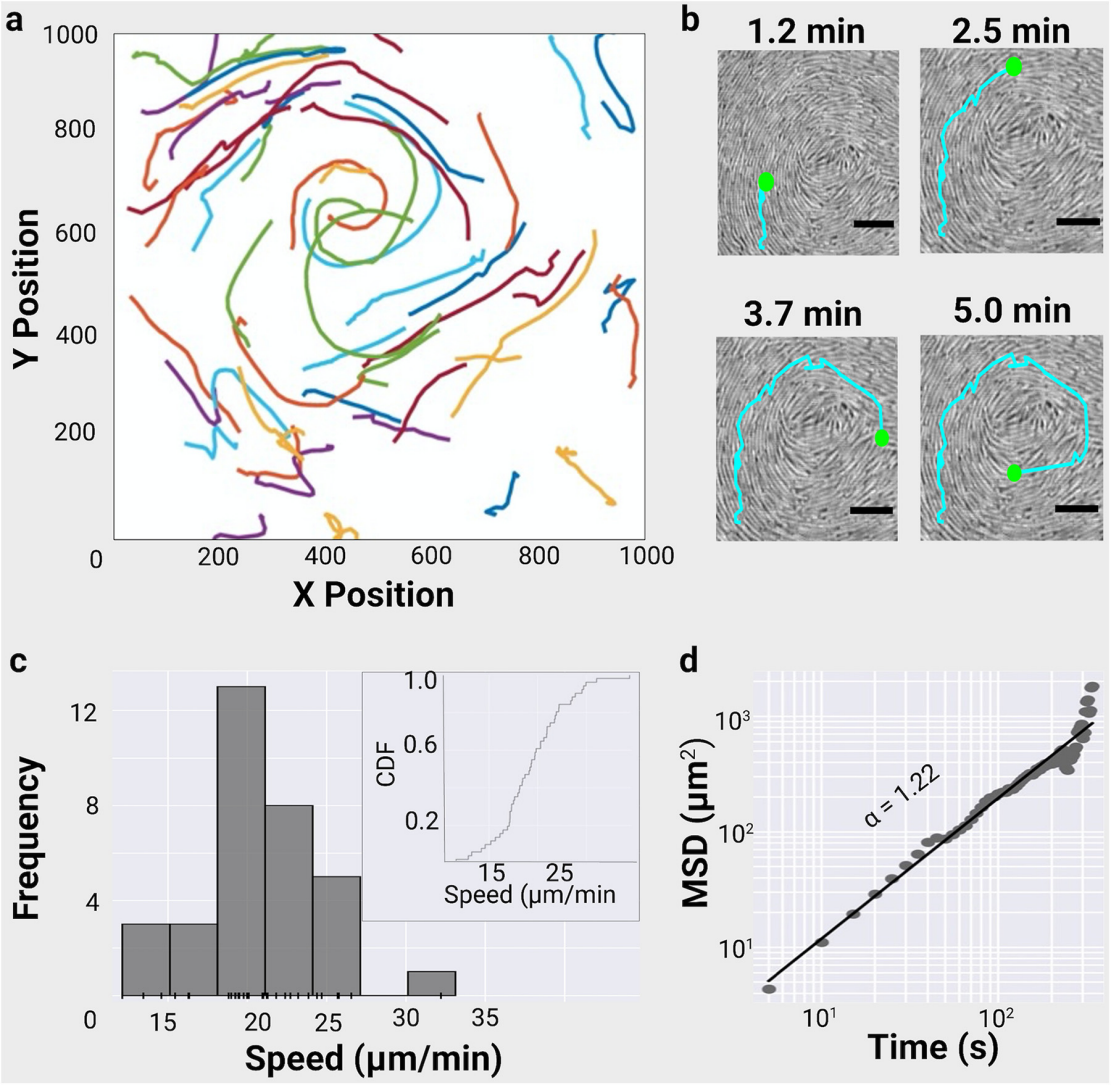
A swarm of *C. gingivalis* actively transports phages. (a) Trajectories from Movie S1 show 33 phages being propelled by a *C. gingivalis* swarm. (b) Time-lapse images of a cropped section of Movie S1 showing a fluorescent lambda phage being transported by a swarm of *C. gingivalis* (gray). The positions of a phage and the trajectory are shown in green and cyan, respectively. Scale bar = 5 μm. (c) A frequency distribution and a rug plot of phage speed. Inset shows the cumulative density function (CDF) of phage speed. (d) Ensemble mean squared displacement of phages from Movie S1 plotted as a function of time. The slope (α = 1.22) of the power-law fit implies that the phages are super diffusive and are actively propelled by a *C. gingivalis* swarm.

Our data show that a swarm of *C. gingivalis*, a bacterium that is abundant in the oral microbiota of healthy humans (14), can actively transport lambda phages over long distances (see Movie S1 in the supplemental material; Fig. 1a and b). The experiments were performed using lambda phages that have yellow fluorescent protein (YFP) attached to their coat protein gpD (21). Lambda phages infect E. coli but cannot infect *C. gingivalis* (see Fig. S1 in the supplemental material).

Phages were transported due to fluid flows generated by the *C. gingivalis* swarm (Movie S1). In most cases, a phage did not bind to a *C. gingivalis* cell (see Movie S2 in the supplemental material). In very rare cases, transient attachment of a phage to a *C. gingivalis* cell was observed (see Movie S3 and Fig. S2 in the supplemental material). A bacterial swarm secretes osmotic agents that form a gradient due to which water moves out from the agar, mixes with surfactants, and forms the swarm fluid (22). Surfactants secreted into the swarm fluid keep the swarming cells wet and help in reduction of water loss from the substratum (23). By the use of microbubbles as tracers, it was shown that the E. coli swarm fluid moves orthogonal to the swarm front’s direction of motion (22). This movement was due to the rotation direction of the E. coli flagellar bundle relative to the cell’s long axis. In the case of gliding bacterium, the mobile adhesin SprB moves along the long axis of the cell. Hence, microbubbles move along the direction of motion of a *C. gingivalis* swarm (15). Tracking showed that phages were transported by *C. gingivalis* along the direction of the swarm’s propagation (Fig. 1a and b) with a mean speed of 20.71 ± 3.96 μm/minute (Fig. 1c), which is also the speed of motion of individual cells in a swarm (15). Changes in mean square displacement (MSD) as a function of time provided a power-law slope of 1.22 (Fig. 1d). The MSD of 98 phages from the 12 additional swarms (see Fig. S3 in the supplemental material) was similar to the value reported in Fig. 1d. The MSD power-law slope (α > 1) indicates that a particle is superdiffusive (actively moving). In contrast, for a diffusive particle, α = 1. Hence, the transportation of phages by a *C. gingivalis* swarm is an active process.

**FIG 2:**
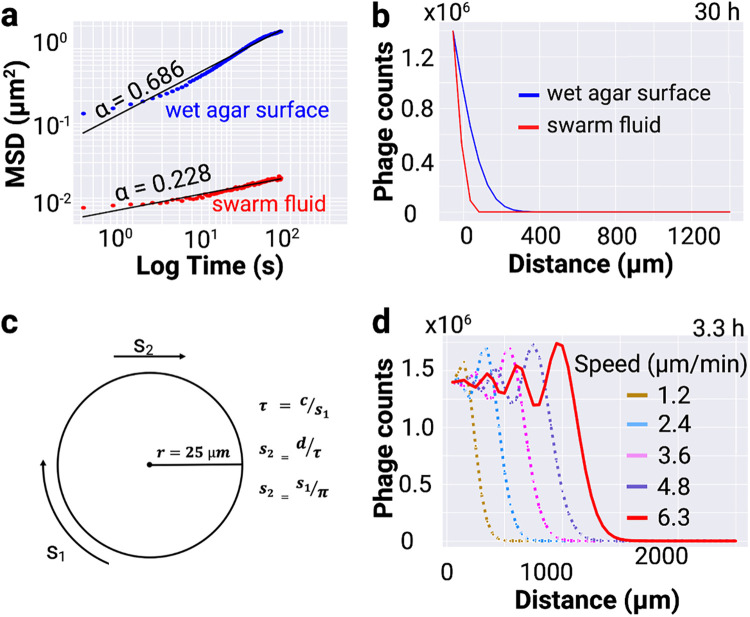
Prediction of the distance travelled by transported phages. (a) Mean squared displacement of phages diffusing in a thin layer of liquid on a wet agar surface (blue) and swarm fluid of bacteria with inhibited motility (red). (b) A prediction of one-dimensional distance covered by phages diffusing in the two fluids described above. (c) A cartoon outlining a vortexing swarm with radius r, circumference c, and vortexing speed s_1_. Using the three equations shown in the inset, linear speed s_2_ is calculated to be 6.3 μm/min. (d) A prediction of one-dimensional distance covered by actively transported phages. Outputs for linear speed ranging from 1.2 μm/min to 6.3 μm/min are shown.

**FIG 3:**
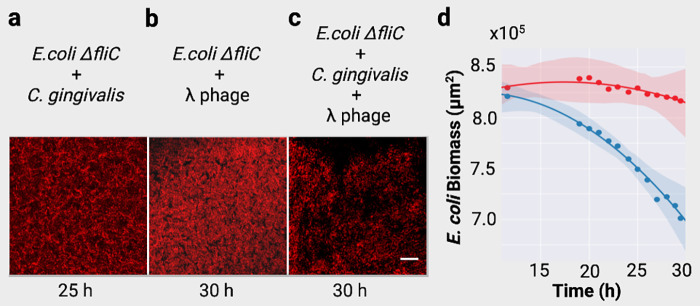
Phage delivery reduces the biomass of the prey. Images of the red fluorescent E. coli colony shown at the end of the timelapses for the control and experimental settings. These images accompany Movie S6 to S8 and Fig. S4 to S8. (a) The control where swarming *C. gingivalis* were inoculated at a distance of 1 mm from the periphery of an E. coli inoculum shows minimal temporal reduction in the biomass of the red fluorescent E. coli colony. (b) The control where λ phage was inoculated at a distance of 1 mm from the periphery of the E. coli inoculum demonstrates minimal temporal reduction in the biomass of the E. coli colony. (c) The experimental condition where a mixture of λ phage and *C. gingivalis* was inoculated at a distance of 1 mm from the periphery of an E. coli inoculum shows a significant temporal reduction of E. coli biomass. (d) Changes in the area of E. coli biomass depicted as a function of time. Dots represent the mean of three biological replicates. The red data points indicate changes in fluorescence from the timelapse captured for b, and the blue shows changes in fluorescence from the timelapse captured in c. Dark lines are second order regression fits, and the light-shaded regions represent the 95% confidence interval.

To test if swarm-mediated phage transportation provides an advantage over diffusion, we performed simulations that are based on Fick’s laws of diffusion (Fig. 2). The predictions of our simulations were also tested experimentally (Fig. 3). To design a control for the experiment described in Fig. 3, it was necessary to find the diffusion constant of phages within a thin liquid layer on the wet agar surface. We inoculated 2 μL of 10^8^ PFU/mL fluorescent lambda phages over an agar surface that was incubated in a 100% relative humidity chamber. Fluorescent phages diffusing in a thin liquid layer on the agar surface were imaged (see Movie S4 in the supplemental material) and their MSD power-law slope was 0.68 (Fig. 2a), which suggests that the phages were subdiffusive. Using the Einstein relation *D* = <Δ*r*^2^>/*n*Δ*t*, we calculated the diffusion coefficient (*D*) of phages on a wet agar surface to be 0.041 μm^2^/second. Similarly, we calculated that the *D* of phages on swarm fluid of bacteria with inhibited motility is 0.228 (Fig. 2a). Here, $\Delta r^{2}\;$ is the change in squared displacement, n is the dimensionality of the diffusion, and Δt is the change in time (4). To measure the distance that phages will diffuse in 30 h at 33°C, we used Fick’s second law of diffusion (4), namely, Δ*C*/Δ*t* = *D*Δ^2^*C*/Δ*x*^2^, where ΔC is the change in concentration, Δt is the change in time, D is the diffusion coefficient, and Δx is the change in distance. We found that within 30 h, phages diffuse about 400 μm on a wet agar surface. In contrast, they diffuse only 50 μm on the swarm fluid of *C. gingivalis* with inhibited motility (Fig. 2b).

*C*. *gingivalis* swarms in a circular fashion wherein new circles propagate from the edge of an initial circular swarm (15). The vortexing speed of the swarm is about 20 μm/minute. Using the three equations shown in Fig. 2c, the linear speed of the swarm was calculated to be 6.36 μm/minute. Using the advection-diffusion equation $\frac{\partial C}{\partial t}=\frac{D^{\partial^{2}C}}{\partial x^{2}}\;-\;v_{d}\frac{\partial C}{\partial x}$, we simulated that *C. gingivalis* can transport phages to a distance of ~1,500 μm in 3.3 h (Fig. 2d). Here, c is the concentration of phages, t is time, x is distance, D is the diffusion coefficient, and $v_{d}$ is advective velocity.

The *in silico* results described in Fig. 2 guided the design of the wet-lab experiments described in Fig. 3. We know that *C. gingivalis* starts swarming about 7 h after initial inoculation on an agar surface. Hence, we started capturing microscopic images to test if phages delivered by *C. gingivalis* can increase the kinetics of E. coli colony disruption 10 h after the initial inoculation. As suggested by the simulation in Fig. 2d, this provided 3 h for phage delivery and 7 h for swarm initiation.

We inoculated a phage-CG mix (phage with *C. gingivalis*, see Materials and Methods) and fluorescent nonmotile E. coli (Δ*fliC* in RP437 background with mCherry) on a wet agar surface. The distance between phage-CG and E. coli spots was 1 mm. Via confocal microscopy, changes in E. coli biomass were measured by capturing images periodically from 10 to 30 h. As controls, similar amounts of either lambda phage or *C. gingivalis* suspension were spotted at a 1-mm distance from the fluorescent E. coli colony. Samples were imaged 500 μm inward from the edge of the E. coli colony. In this region, minimal reduction in E. coli biomass was observed in the control (Fig. 3a and b; see Movie S6 in the supplemental material). This finding implies that artificial fluid flows that might occur during sample preparation and addition of cover glass cannot deliver phages to this region. No change in E. coli biomass was observed when *C. gingivalis* alone was spotted at a 1-mm distance from the E. coli colony (see Fig. S6 and S7 and Movie S8 in the supplemental material). This control showed that interbacterial competition due to metabolites or physical forces do not play a role in the reduction of biomass of E. coli. In contrast, when phages were actively transported by *C. gingivalis*, the E. coli biomass was reduced at a 10 times higher rate than the controls (Fig. 3a, b, and c; see Fig. S4 and Movie S7 in the supplemental material).

**FIG 4:**
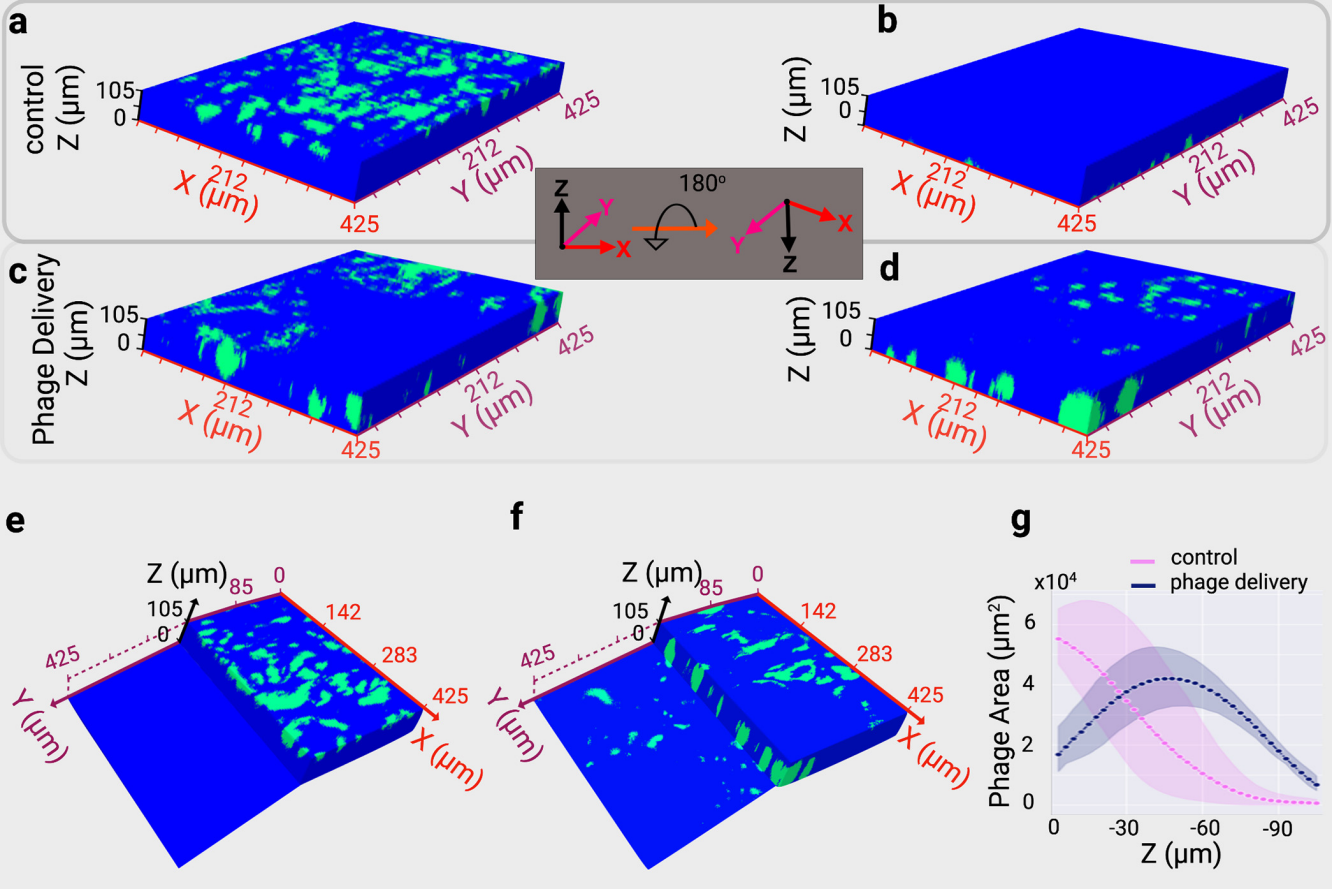
Transportation by *C. gingivalis* increases the three-dimensional phage delivery within a mature E. coli biofilm. Phages are in green, and blue depicts the E. coli biofilm. (a) Top view of the control E. coli biofilm with only λ phage added. (b) Bottom view of the same biofilm. These images are taken from Movie S9. (c) Top view of the experimental E. coli biofilm with the addition of λ phage and unlabeled *C. gingivalis*. (d) Bottom view of the same biofilm. These images are taken from Movie S10. (e) A slice of the biofilm from a and b, allowing a partial view of the top and bottom layer for the control. (f) A slice of the biofilm allowing a partial view of the top and bottom layer of the biofilm from c and d for the experimental setting. (g) The location of phages along the z-axis show that after delivery by *C. gingivalis*, phages are found in all regions within the biofilm, with maximum area coverage present at a depth of ~50 μm. Whereas the location of naturally diffusing λ phages in the control is clustered primarily within the top regions. Connected dots represent the mean from three biological replicates. The light-shaded regions represent the 95% confidence interval.

Furthermore, we hypothesized that the fluid flows generated by a swarm might deliver phages to biofilms that are inherently protected from phage infection. Recently, it was demonstrated that a polymeric mesh generated by curli fiber creates a physical barrier that protects a biofilm from phage infection (3). The curli fiber-producing E. coli strain WR3110 (3) was used, and the production of curli fiber under our experimental conditions was detected using an amyloid dye thioflavin, S^44^ (see Fig. S9 in the supplemental material). Furthermore, an mCherry-containing plasmid was introduced into the curli fiber-producing E. coli strain. As a control, we inoculated phages on top of a curli fiber-producing fluorescent E. coli biofilm, and we found that most phages were concentrated at the top layer of the biofilm (Fig. 4a to c). In contrast, effective phage penetration was observed when phages were transported by a *C. gingivalis* swarm to a curli fiber-producing fluorescent E. coli biofilm. Strikingly, actively transported phages even reached the bottom (105-μm depth) of the biofilm (Fig. 4d to f) with the largest number of phages found at a depth of about 50 μm from the top of a biofilm that has a total depth of 105 μm (Fig. 4g). These data show that active phage transportation can bypass the physical barriers present within a biofilm and suggest a novel role of motility in the development of a polymicrobial community.

**FIG 5:**
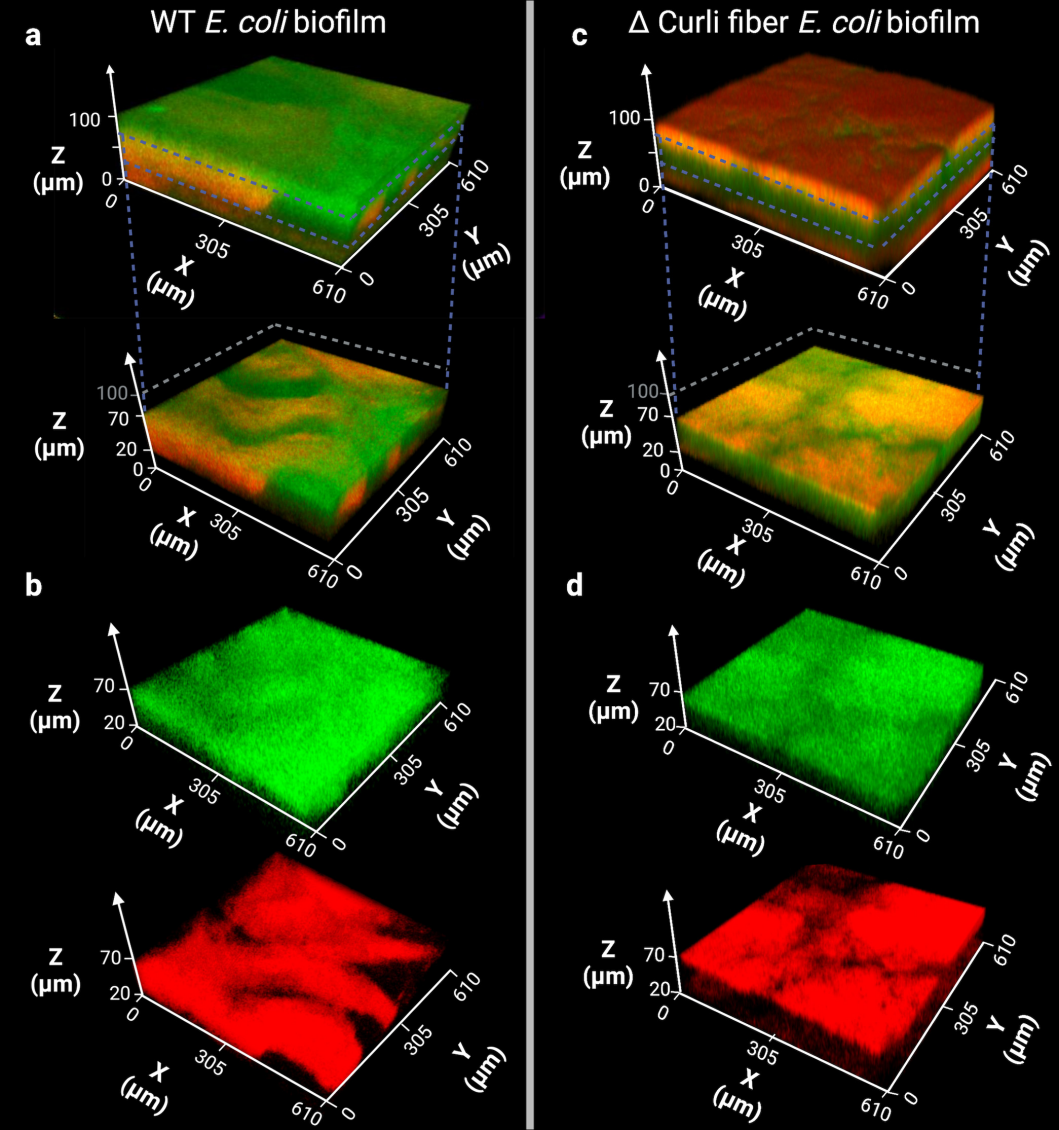
Fluorescence *in situ* hybridization of *C. gingivalis* swarms on E. coli biofilms. (a) A 3D rendering of curli fiber-containing E. coli biofilms grown and spotted with *C. gingivalis* swarms. Green fluorescence indicates *C. gingivalis*, stained with a fluorescently labeled 16S rRNA probe targeting *Capnocytophaga* sp., and red fluorescence indicates mCherry-tagged E. coli. (Top) Shows the entire 105-μm-deep biofilm, covered by a layer of *C. gingivalis*, which was segmented in the second row to display the inner sections of the biofilm between 20 and 70 μm. In this region of the biofilm, E. coli cells were displaced by what appear to be *C. gingivalis* tunnels. (b) The individual channels of the segmented biofilm shown in a. (c) In the absence of curli fibers, *C. gingivalis* no longer forms a layer on top of the biofilm and instead penetrates into the middle layers of the biofilm. (d). Individual channels of the segmented biofilm shown in c.

In order to learn how a *C. gingivalis* swarm disrupts the physical barrier created by a polymeric mesh of curli fiber, *C. gingivalis* cells were introduced along the circumference of a curli fiber-producing fluorescent E. coli biofilm. Swarming was allowed for 24 h, and *C. gingivalis* cells were stained by fluorescence *in situ* hybridization (FISH) using a probe specific to the 16S rRNA of *Capnocytophaga* sp. (see Fig. S10 in the supplemental material). *C. gingivalis* formed tunnel-like structures within a 50-μm-thick zone of the curli fiber-producing E. coli biofilm (Fig. 5a and b; see Fig. S11a and c in the supplemental material). Also, the *C. gingivalis* swarm appeared to physically displace the E. coli cells which might help them maneuver through the curli fibers. The total height of the E. coli biofilm was 105 μm, and the zone preferred by *C. gingivalis* spanned from a 20-μm depth to a 70-μm depth from the top of the E. coli biofilm. This zone is in a similar location as the maximum area of phages within the biofilm structure observed in Fig. 4g.

In a control where a non-curli-fiber-producing E. coli biofilm was used, less distinct spatial structures were observed and *C. gingivalis* completely penetrated the non-curli-fiber-producing E. coli biofilm (Fig. 5c and d; Fig. S11 b and d). However, when introduced to an E. coli biofilm that produced curli fibers, *C. gingivalis* created a thick layer on top of the biofilm and formed tunnel-like structures inside the E. coli biofilm. A lower abundance of *C. gingivalis* was detected throughout the curli fiber-producing E. coli biofilm.

Single cells of *C. gingivalis* and other closely related bacteria move in a screw-like fashion (15, 16). It is possible that a swarm of *C. gingivalis* moves through and expands the gaps in the mesh of curli fiber. This process could effectively lead to “drilling of tunnels” in a biofilm and might drive a stream of fluid that carries phages. A *C. gingivalis* swarm contains multiple motile cells, and the data presented above demonstrate that the collective motion of gliding cells enables phage transportation.

## DISCUSSION

We report that the active transportation of phages by a bacterial swarm increases the frequency of interaction between the predator and the prey. This increase enhances the clearance of the biomass of an E. coli colony. The transporter *C. gingivalis* forms tunnel-like structures within an E. coli biofilm and delivers phages to previously inaccessible regions of the biofilm.

While our work improves the fundamental understanding of microbial ecology, in the future, it can also be applied to improve the pharmacokinetics of phage therapy. Topical application of phage is the preferred method for treating burn wound infection (24) and chronic ear infection (otitis media) (25). In an *in vitro* setting, our data demonstrate an improved delivery of topically applied phages within an E. coli biofilm. Whether such a phage transporter might provide an alternative approach for improved delivery of all phages in an *in vivo* setting remains to be tested.

Bacterial motility machineries are typically regulated by chemotaxis (26); however, very little is known about the chemotaxis of *Capnocytophaga* sp. and other related gliding bacteria. Bacteria that glide using the T9SS do not have the flagellar machinery. *Capnocytophaga* sp., which has the T9SS, carry putative sensors and regulators, but it does not have the genes that encode the chemotaxis network that controls the flagellar motor in other bacteria. Swarms of *Capnocytophaga* sp. seem to move in a directional manner, but it is unknown if this directionality is due to a novel chemosensory pathway that might control the rotational direction of T9SS. In theory, using chemotaxis to control the direction of a swarm could help deliver active phages to chronic biofilms that grow in hard-to-reach areas. We observed that most of the tunneling by *C. gingivalis* happens at depths between 20 and 70 μm within the E. coli biofilm. Future research may reveal whether the chemotaxis of the transporter guides the predator to preferentially localize within the biofilm of its prey.

Swarming *C. gingivalis* generate an active flow within their swarm fluid (15), and we find that phages can travel along this fluid flow. In another scenario, if *C. gingivalis* could bind to specific phage proteins, the steric hindrances might impede the screw-like motility of the gliding transporter cell. Thereby, the overall efficiency of transport might decrease. Since nonhost phages do not need to bind to *C. gingivalis*, there is a possibility of the transport of diverse phages via this mechanism. On the flip side, the phages that could bind to *C. gingivalis* might be small enough to not produce significant steric hinderances. Currently, no host phages are known for *Capnocytophaga* sp., but further examination of phage diversity of the human oral cavity might help answer how host-transported phages compare with non-host-transported phages. The natural habitat of bacteria of the *Capnocytophaga* genus is the human oral microbiota, and they are abundantly enriched in human supragingival and subgingival biofilms (14). Why is the *Capnocytophaga* genus abundant in the oral microbiota? What is their role in human oral polymicrobial biofilms? Why do they have robust gliding motility? These questions beg for answers. We reported previously that they transport other nonmotile microbes of the oral microbiota (15). Now, we report that they can also transport phages. These phages do not need to adhere to *Capnocytophaga* cells; rather they flow along the swarm fluid. In the oral microbiota, several other nonhost phages might get propelled by *Capnocytophaga* swarms. While a lot needs to be learnt about the physiology and ecology of the *Capnocytophaga* genus, our experiments suggest that public transportation might be one of the major contributions of the *Capnocytophaga* genus toward the development of the spatial structure of the human oral microbiota.

## MATERIALS AND METHODS

### Growth of *C. gingivalis*.

Motile *C. gingivalis* ATCC 33624 was grown on Trypticase soy yeast (TSY) agar (30 g/L Trypticase soy broth, 3 g/L yeast extract, and 1.5% Difco Bacto agar), as described previously (15). Growth was carried out in a CO_2_-rich anaerobic environment with 100% relative humidity. Briefly, inoculated agar plates were placed in an AnaeroPack system jar (Mitsubishi Gas Chemical) with candles, an ignited sheet of Kimtech paper, and a beaker full of water. The box was sealed and incubated at 37°C for 48 h. These conditions are optimal *C. gingivalis* motility. A *C. gingivalis* suspension was prepared by scraping 27 μg of *C. gingivalis* (wet weight) from a plate and resuspending it in 50 μL of sterile and double-distilled water.

### Preparation of phage lysate.

Fluorescent phage λ_LZ641_ (gift from Lanying Zeng, Texas A&M University) used in this study contains a mixture of wild-type head stabilization protein gpD and a fusion of gpD with YFP (27). E. coli strain MG1655 was grown in Luria-Bertani (LB) agar at 37°C and in LB broth with overnight shaking at 37°C. A total of 100 μL of the E. coli culture was mixed with 100 μL of either 10^−6^ or 10^−7^ dilution of a 10^8^ PFU/mL lysate of λ_LZ641_. After incubation at room temperature for 15 min, the mixture was added to 4 mL top agar (LB with 0.7% agar) (50°C), gently mixed, and poured onto LB agar. After overnight incubation at 37°C, a plaque containing top agar was resuspended in 1.2 mL of sterile water. After incubation for 4 h at room temperature, the slurry was centrifuged at 1,900 relative centrifugal force (rcf) for 5 min. The supernatant was passed through a 0.2-μm filter and either kept at 4°C for immediate use or at −20°C for long-term storage. For the plaque assay shown in Fig. S1, a method similar to the one described above was used with one notable exception—instead of LB, TSY was used for both E. coli and *C. gingivalis*.

### E. coli growth conditions.

E. coli was grown on LB agar and broth. The plasmid pBT1 mCherry (a gift from Michael Lynch, Addgene plasmid no. 65823) was inserted via electroporation into nonmotile E. coli (Δ*fliC* in RP437 background). Cells containing pBT1-mCherry were cultured on LB agar with 100 μg/mL ampicillin at 37°C. A single colony was inoculated in 3 mL LB broth with ampicillin at 37°C and was grown overnight with shaking. Subsequently, a day culture was prepared by adding 300 μL of the overnight culture to 30 mL of LB broth with ampicillin, and cells were grown to mid-log phase at 37°C while shaking. The day culture was used for subsequent experimentation.

Curli fiber-producing E. coli WR3110 strain (3) (gift from Knut Drescher, University of Basel) was grown on LB agar at 37°C. Like the method described above, plasmid pBT1 mCherry was inserted into E. coli WR3110. To test curli fiber production by a biofilm of E. coli WR3110, it was grown on TSY agar supplemented with an amyloid dye thioflavin S^44^ (40 g·ml^−1^) (28) for 48 h at 28°C. After incubation, mature biofilms were imaged using a Zeiss LSM 880 confocal microscope (Oberkochen, Germany), and z-stack images (3-μm slices) of the E. coli biofilm were obtained. To visualize the distribution of curli fibers within the biofilm, the maximum intensity projection from z-stacks was reconstructed using the Zeiss Zen software.

### Imaging and analysis of phage transportation.

A total of 10 μL of fluorescent λ_LZ641_ phage lysate was added to 20 μL of a C. *gingivalis* suspension, and we refer to this solution as the phage-CG mix. Next, 2 μL of the phage-CG mix was spotted onto a TSY agar pad on a slide and incubated for 5 min at room temperature. Subsequently, a coverslip was placed, and the edges were sealed using beeswax (Aqua Solutions, Inc.; lot no. 730701). The slides were incubated at 37°C for 3 h under conditions optimum for *C. gingivalis* motility (described above).

Images were captured with the Zeiss LSM880 confocal microscope, and phages were tracked using an ImageJ plugin TrackMate (29). The two-dimensional position coordinates of each phage particle were exported from ImageJ in an .xml format. The .xml files were imported into MATLAB (MathWorks, Natick, MA), and converted to a .csv file. The .csv file was imported into Python and converted to DataFrame. The mean speed of each phage particle was calculated using a custom MATLAB script, and the mean square displacement (MSD) was calculated using a custom Python script based on Trackpy (30).

To test if phages attach to individual *C. gingivalis* cells, 50 μL phage-CG mix was injected into a tunnel slide. The tunnel slide was incubated at room temperature for 5 min, and 50 μL of 10% methyl cellulose was added as described previously (15). Motile *C. gingivalis* cells on a cover glass and fluorescent phages were imaged by a Zeiss LSM880 confocal microscope.

### Measurement of the diffusion coefficient of phage particles and simulation of phage delivery.

To calculate the diffusion coefficient of phages on a wet agar surface, 2 μL of 10^8^ PFU/mL λ_LZ641_ was spotted onto a freshly prepared TSY agar pad and incubated for 5 min at room temperature. Subsequently, a coverslip was placed, and the edges were sealed using beeswax. Time-lapse images of phages diffusing within a thin liquid layer between the agar and cover glass were captured with the Zeiss LSM880 confocal microscope. By use of custom Python scripts, the ensemble MSD of phage particles was calculated.

In order to calculate the diffusion coefficient of phages diffusing in swarm fluid of bacteria with inhibited motility, a 48-h-old culture of *C. gingivalis* was treated with 10 μM carbonyl cyanide-*m*-chlorophenylhydrazone (CCCP), an uncoupler which disrupts the proton motive force and stops T9SS-driven motility (15, 31). CCCP-treated *C. gingivalis* was used to prepare a phage-CG mix which was spotted onto a TSY agar pad. As described above, ensemble MSD of phage particles diffusing in swarm fluid of nonmotile *C. gingivalis* was calculated.

To find how far apart E. coli and phage-CG mix should be spotted onto a TSY agar plate, we performed simulations of phages diffusing on wet agar surface and swarm fluid of bacteria with inhibited motility. Additionally, the distance traveled due to active transportation of phages by a swarm of *C. gingivalis* was also simulated.

### Change in biomass of the E. coli colony after phage delivery.

A day culture of nonmotile E. coli (Δ*fliC* in RP437) containing plasmid pBT1 mCherry was pelleted and resuspended in 250 μL of sterile water. A total of 2 μL of the suspension was spotted onto a petri dish containing TSY agar. Next, 2 μL of phage-CG mix was spotted at 1,000 μm from the E. coli spot. The plate was incubated for 10 h under conditions optimum for *C. gingivalis* motility (described above). After incubation, a circular slab of agar (around 35-mm diameter) surrounding the two colonies was cut with a scalpel and transferred onto the lid of a smaller petri plate (35 by 10 mm). A coverslip was placed, and the edges were sealed using beeswax. With a Zeiss LSM880 confocal microscope, time-lapse images were obtained for the next 21 h. The interval between each captured image was 1 h. The area of the E. coli colony was calculated using the Python library scikit-image (32). The change in biomass of E. coli after phage delivery was determined via a custom Python script.

### Assay of phage delivery within a mature E. coli biofilm.

Curli fiber-producing E. coli strain WR3110 with plasmid pBT1 mCherry was grown on a TSY agar petri plate for 48 h at 28°C. The lower temperature maximizes curli fiber production and biofilm formation (33). After incubation, a 50-μL *C. gingivalis* suspension was pipetted around the circumference of the mature E. coli biofilm, and the plate was transferred for a 14-h incubation under conditions optimum for *C. gingivalis* motility (described above). After incubation, 20 μL of 10^5^ PFU/mL λ_LZ641_ was spotted on the biofilm, and the plate was incubated for another 10 h under conditions optimum for *C. gingivalis* motility (described above). After incubation, z-stack images (3-μm slices) of the E. coli biofilm were obtained using a Zeiss LSM 880 confocal microscope. The z-stack images were analyzed via custom Python scripts that use the scikit-image library. Three-dimensional images and movies of phage penetration were reconstructed from the z-stack images via a Python library Mayavi (34). All data and custom scripts are freely available online at https://github.com/krNichith/Phage_Delivery.git.

### Fluorescence *in situ* hybridization.

A total of 5 μL of a day culture of curli fiber-producing E. coli WR3110 with plasmid pBT1 mCherry was incubated for biofilm production on a TSY agar pad for 48 h at 28°C. Next, 50 μL of a *C. gingivalis* suspension was spotted around the circumference of the E. coli biofilm and incubated for 24 h under conditions optimum for *C. gingivalis* motility (described above). After the incubation, a thin section of the top layer of agar was sliced and placed on a silane-coated glass slide (Electron Microscopy Sciences, Hartford, PA; catalog no. 63411-01) with three GeneFrame chambers (Thermo Scientific, Pittsburgh, PA; catalog no. AB0577) stacked on top of the other. A total of 250 μL of phosphate-buffered saline (PBS; pH 7.1) with 4% of fixing agent paraformaldehyde was poured on the biofilm, and the slide was placed in an empty petri dish. A humidity chamber was created by adding 25 mL water to a glass Tupperware box (6″ by 4″ by 2″). The petri dish containing the slide was incubated for 2 h at 4°C in the humidity chamber. After incubation, the biofilm was gently washed with 250 μL of PBS. Subsequently, 250 μL of 1 mg/mL lysozyme in 20 mM Tris-HCl (pH 7.5) was added to the GeneFrame chamber and incubated at 37°C for 30 min in the humidity chamber. The lysozyme solution was replaced with a 250 μL hybridization buffer which contained 20 mM Tris-HCl (pH 7.5), 0.9 M NaCl, 20% formamide, 0.01% SDS, and 250 nM probe fluorescently labeled with tetrachlorofluorescein (TET). The probe targets the 16S rRNA of *Capnocytophaga* spp. (5′-TCA GTC TTC CGA CCA TTG-3′) (14, 35), and it was manufactured by Biosearch Technologies, Petaluma, CA. The samples were incubated at 46°C for 4 h in a modified humidity chamber where 20% formamide was used instead of water. The hybridization solution was removed, and biofilms were washed in 250 μL wash buffer (20 mM Tris-HCl [pH 7.5], 215 mM NaCl, and 5 mM EDTA) for 15 min at 48°C. The wash solution was removed, and biofilms were mounted in VectaShield vibrance antifade mounting solution (Fisher Scientific, Pittsburgh, PA; catalog no. H-1700-10) for a minimum of 1 h at 4°C prior to imaging. The z-stack images (3-μm slices) were acquired using a Zeiss LSM 880 confocal microscope and were analyzed using ImageJ. The three-dimensional images and movies of phage penetration were reconstructed via ImageJ. All of the final figures were created with BioRender.

### Data availability.

Example data sets and custom data analysis scripts are freely available online on GitHub at https://github.com/krNichith/Phage_Delivery.git.
