# Supplementary material for: Bacterial Swarm-Mediated Phage Transportation Disrupts a Biofilm Inherently Protected from Phage Penetration
Source: Microbiol Spectr. 2023 Jun 26;11(4):e00937-23. doi: 10.1128/spectrum.00937-23 (PMC10434198; doi:10.1128/spectrum.00937-23)
Supplement: Supplemental file 1 — Supplemental material. Download spectrum.00937-23-s0001.pdf, PDF file, 9.4 MB [file spectrum.00937-23-s0001.pdf]

Supplementary Materials

Bacterial swarm-mediated phage transportation disrupts a biofilm inherently protected from phage penetration

Authors. Nichith K. Ratheesh, Amanda M. Zdimal, Cole A. Calderon, and Abhishek Shrivastava*

*Corresponding author. Email: ashrivastava@asu.edu

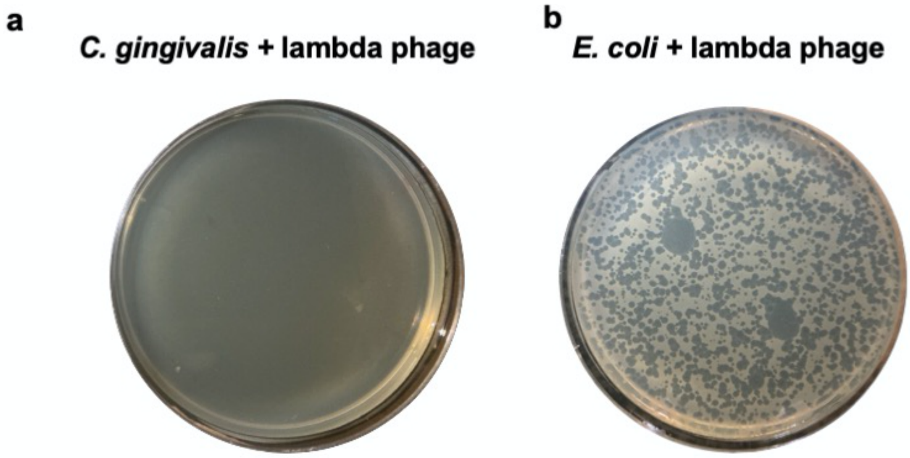

Fig. S1. **a,** A plaque assay shows that λ_{LZ641} does not infect *C. gingivalis*. **b,** As a control, a plaque assay shows infection of *E. coli* by λ_{LZ641}.

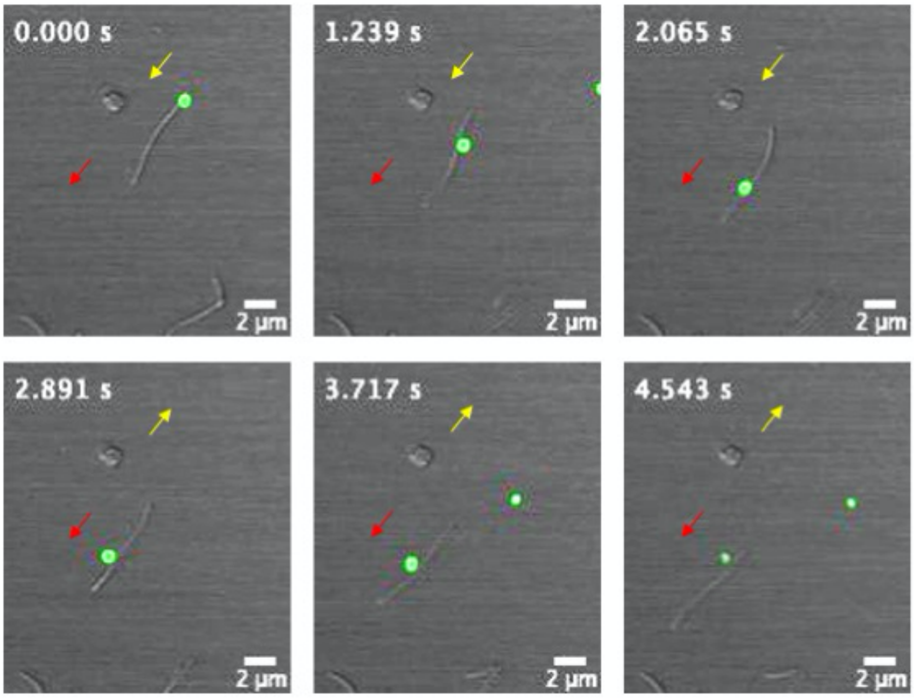

Fig. S2. A time-lapse image of a phage particle being transiently propelled along the surface of a *C. gingivalis* cell. Yellow arrow indicates the direction of motion of phage and the red arrow indicates the direction of motion of a *C. gingivalis* cell. The pattern for cell-surface motion of phage is similar to previously observed motion of the cell-surface adhesin SprB.

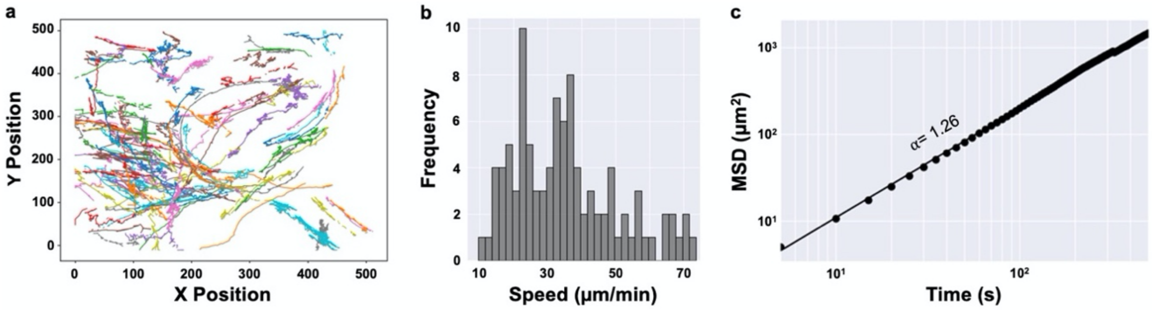

Fig. S3. a, Combined trajectories from 12 movies with 98 phages propelled by different *C. gingivalis* swarms. b, A frequency distribution of the speed at which the 98 phages are transported by *C. gingivalis*. As described previously(15), the speed is proportional to the number of layers within a swarm. The number of layers within the 12 swarms varies. Hence, we see multiple peaks. c, Ensemble mean squared displacement of the 98 phages plotted as a function of time. The slope (α = 1.26) of the power-law fit implies that the phages are super diffusive and are actively propelled by a *C. gingivalis* swarm.

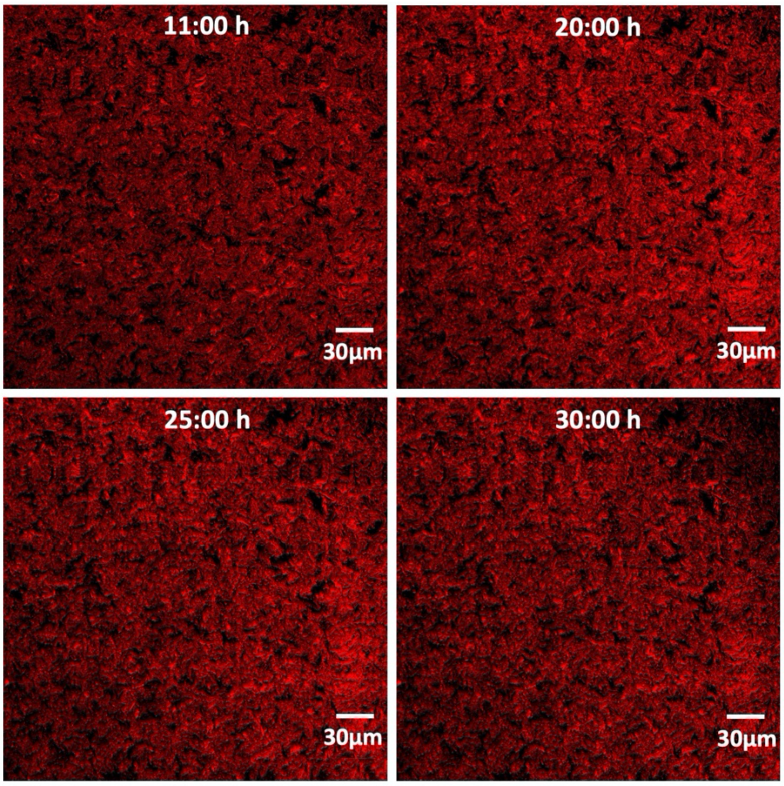

Fig. S4. Images of an *E. coli* colony shown at different time points. Here, phages were spotted 1500 μm away from the imaged spot and were diffusing within a thin liquid layer of wet agar (Movie 6).

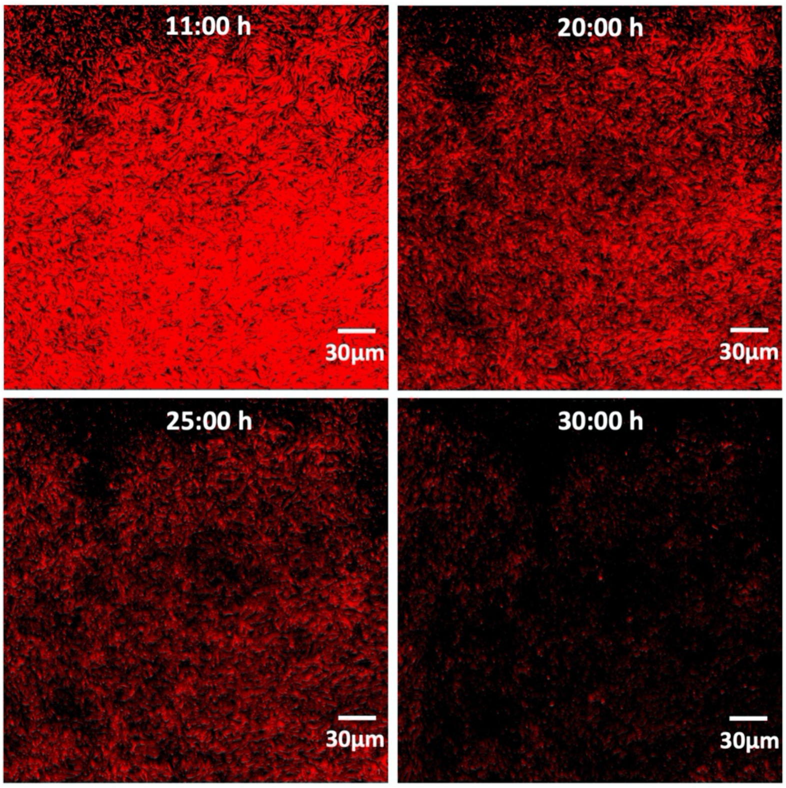

Fig. S5. Images of an *E. coli* colony shown at different time points. Here, phage-CG mix was spotted 1500 μm away from the imaged spot and phages were actively delivered by a swarm of C. *gingivalis* (Movie 7).

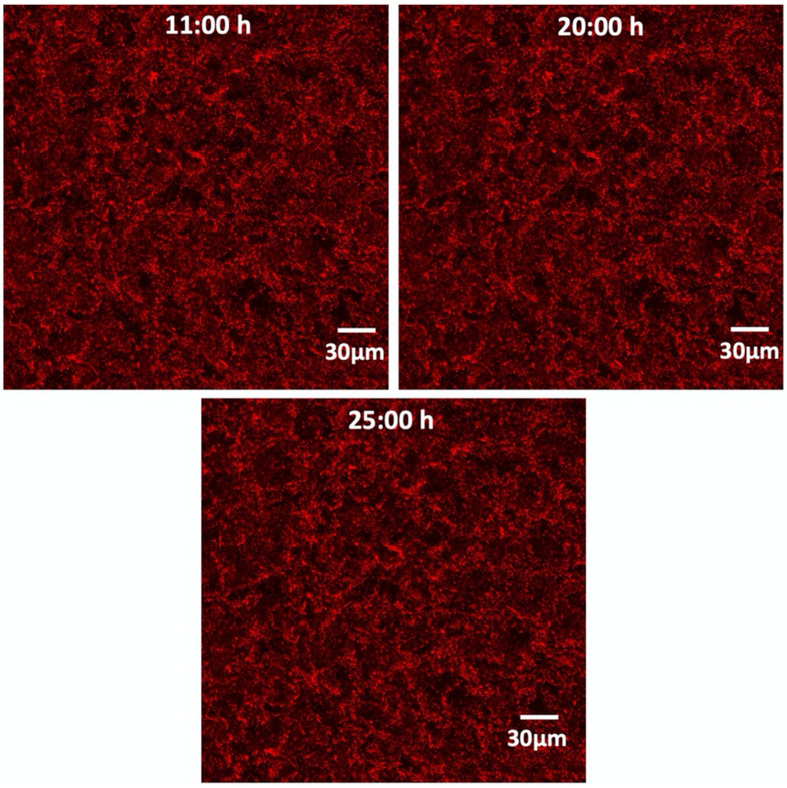

Fig. S6. Images of an *E. coli* colony shown at different time points. Here, only *C. gingivalis* cells were spotted 1500 μm away from the imaged spot. No phages were present (Movie 8).

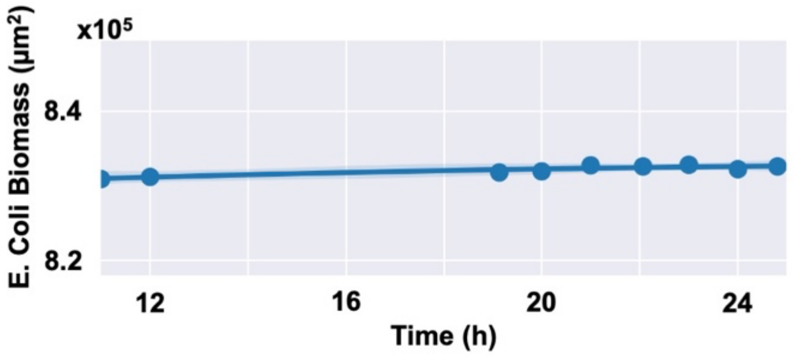

Fig. S7. Changes in the area of the *E. coli* biomass depicted as a function of time when only *C. gingivalis* invades an *E. coli* colony (same scenario as Fig S6). These measurements are from Movie 8. This control shows that there is no change in *E. coli* biomass due to the presence of only *C. gingivalis*.

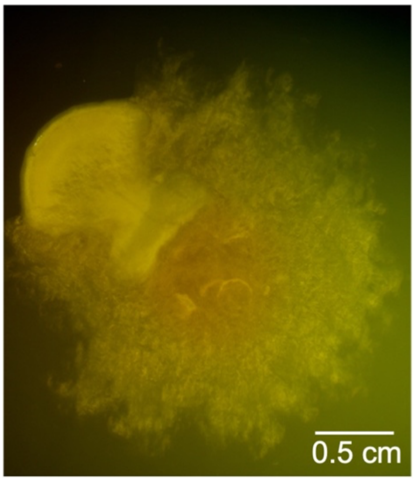

Fig. S8. A colony image taken at 30 hours after inoculation showing that *C. gingivalis* (rough) swarms over *E. coli* (smooth).

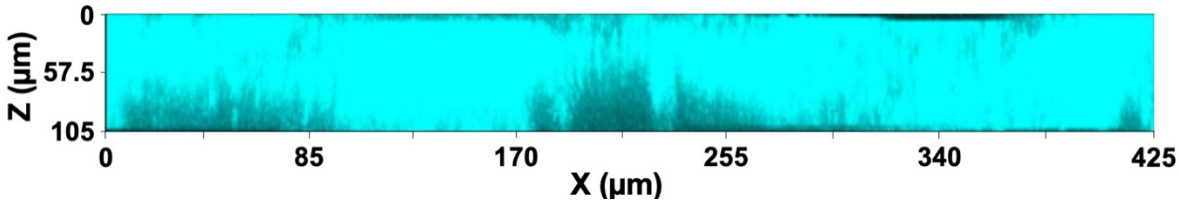

Fig. S9. A maximum intensity projection (cyan) of stained curli fiber from z-stack images of a 72 h old *E. coli* biofilm.

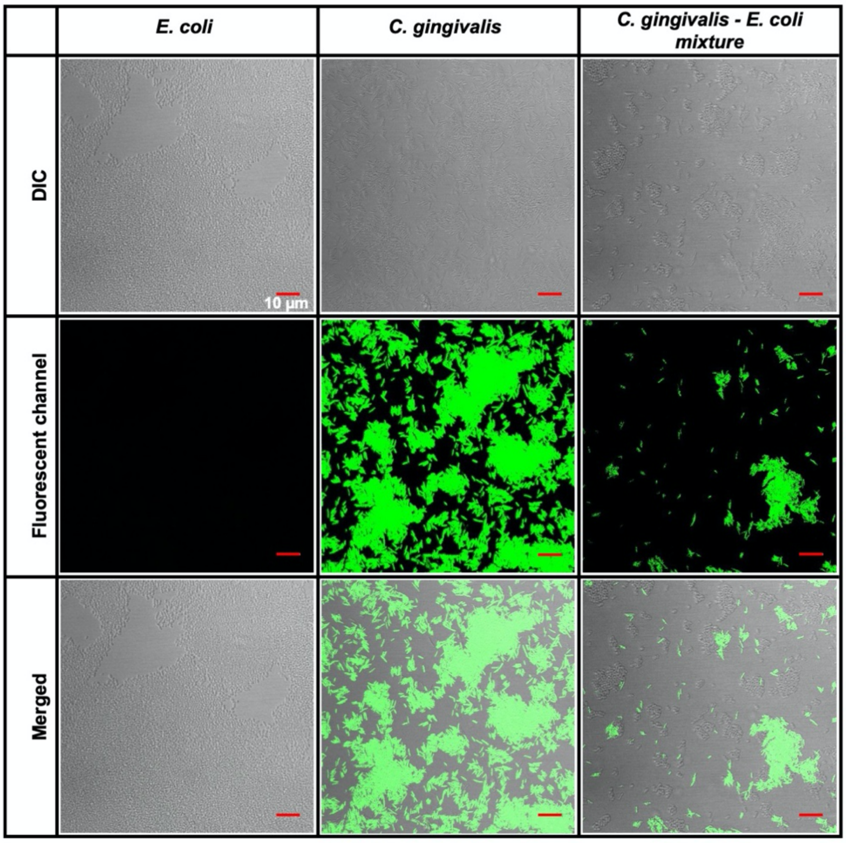

Fig. S10. Fluorescence *in situ* hybridization control for probe specificity. Hybridization was performed using a probe targeting the 16S rRNA of *Capnocytophaga* sp. Pure cultures of *E. coli* (left panel) and *C. gingivalis* (middle panel), as well as a mixed culture of *E. coli* and *C. gingivalis* cells (right panel) were used as a specificity control. The *Capnocytophaga* sp. probe (green fluorescence) bound exclusively to *C. gingivalis* and *E. coli* remained unstained. Individual images of the DIC and fluorescent channels along with the merged view of the two channels are shown.

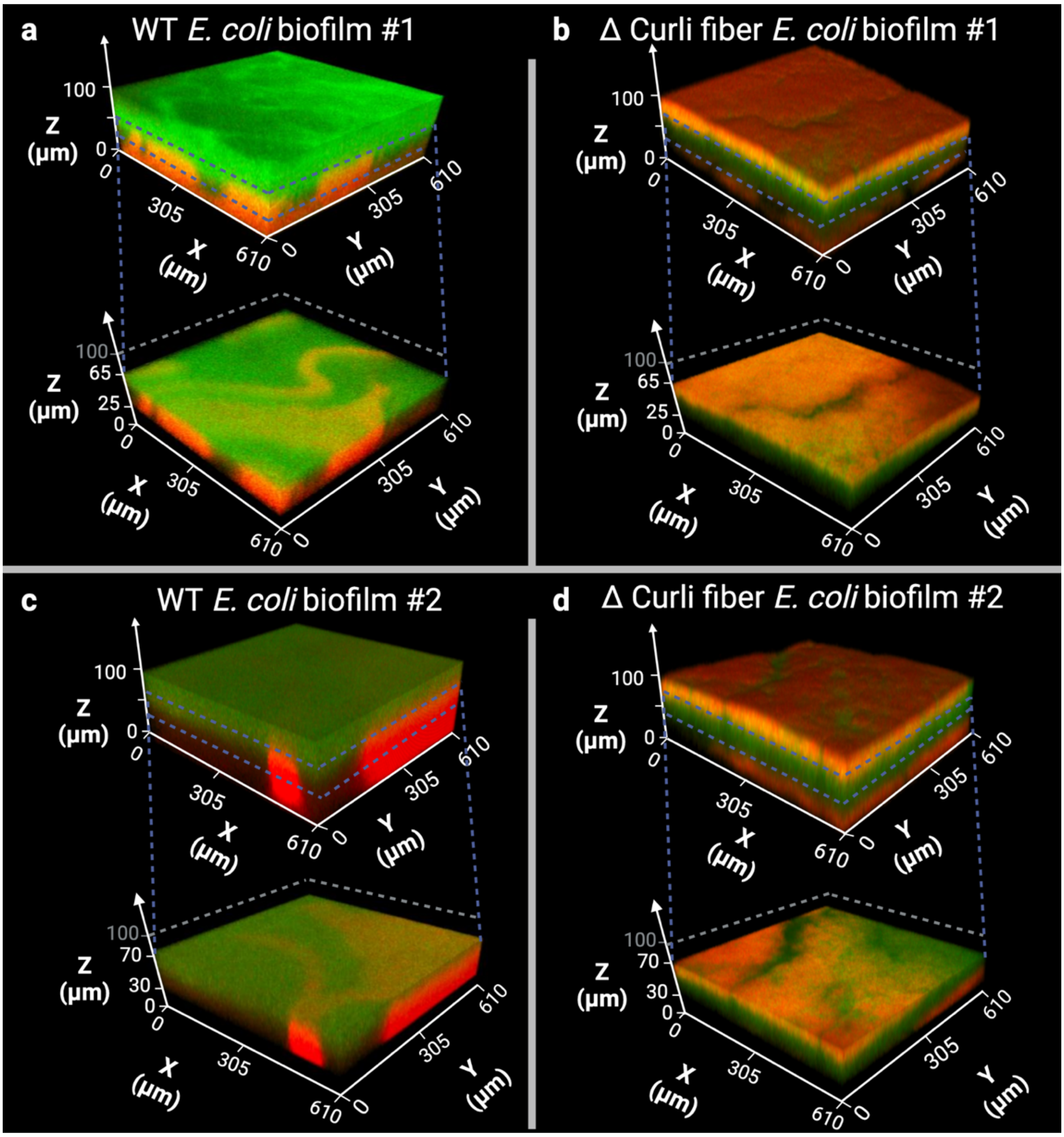

Fig. S11. Images of two additional biofilms grown and stained similar to the biofilm shown in Fig. 5 of the main text.

Supplemental Movies.

Movie 1. Fluorescent lambda phage (green) being actively transported by a swarm of *C. gingivalis* (gray).

Movie 2. An example where lambda phage (green) does not adhere to a motile *C. gingivalis* cell.

Movie 3. An example where a lambda phage (green) transiently attaches to *C. gingivalis* cell and is transported as cargo.

Movie 4. Fluorescent lambda phage (green) diffusing in the thin layer of liquid on the surface of wet agar incubated with 100% relative humidity.

Movie 5. Fluorescent lambda phage (green) in the swarm fluid of a *C. gingivalis* swarm that has stopped swarming.

Movie 6. Time-lapse images of a fluorescent *E. coli* colony (red) in the diffusion control where phages are in a thin layer on a wet agar surface spotted next to the *E. coli* biofilm. Minimal changes in fluorescence are observed, suggesting the inability of naturally diffusing phages to penetrate and disrupt *E. coli* cells deep within the biofilm.

Movie 7. Time-lapse image of a fluorescent E. *coli* colony (red) in the experiment where phages are delivered by C. *gingivalis* swarms. Significant changes in fluorescence are seen, which indicate enhanced phage penetration of the *E. coli* biofilm via *C. gingivalis* swarm, and subsequent phage killing of *E. coli* cells within the biofilm.

Movie 8. Time-lapse image of a fluorescent *E. coli* colony (red) in the control that only has *C. gingivalis*.

Movie 9. A 3-dimensional view of phage (green) penetration in a 425 μm X 425 μm X 105 μm cross section of the biofilm. Phages were diffusing in a curli fiber producing *E. coli* biofilm.

Movie 10. A 3-dimensional view of phage (green) penetration in a 425μm X 425μm X 105μm cross section of the biofilm. Phages were actively delivered to a curli fiber producing *E. coli* biofilm by a swarm of *C. gingivalis.*
